# Crossed Cerebellar Diaschisis in Thalamic Lymphoma on ^18^ F-FDG PET/CT

**DOI:** 10.1055/s-0042-1757253

**Published:** 2022-09-09

**Authors:** Amit Bhoil, Igor RacuAmoasii, Sobhan Vinjamuri

**Affiliations:** 1 Department of Nuclear Medicine, The Royal Liverpool University Hospital NHS Trust, Liverpool, United Kingdom; 2 Haemato-oncology Diagnostic Service, Liverpool Clinical Laboratories, Liverpool, United Kingdom

**Keywords:** PCNSL, thalamic lymphoma, DLBCL, crossed cerebellar diaschisis (CCD), PET/CT

## Abstract

Primary central nervous system lymphomas (PCNSLs) are extranodal variant forms of non-Hodgkin lymphoma arising within the brain parenchyma, leptomeninges, or spinal cord. PCNSL can present with varied neurological symptoms and imaging findings, making diagnosis without biopsy difficult. PCNSLs are highly aggressive, causing rapid deterioration, but are responsive to chemotherapy and radiotherapy making early diagnosis important.

Crossed cerebellar diaschisis (CCD) is mostly seen with cerebral cortex vascular insults and is rarely reported with thalamic lesions and even rarer with thalamic lymphoma. However, CCD has also been described in other brain tumors (including primary glioma), chronic subdural hematoma, congenital insults, intracranial infections, and various dementia subtypes.

We present a rare case of thalamic lymphoma evaluated with positron emission tomography/computed tomography that showed hypermetabolism of thalamus and associated hypometabolism in ipsilateral cerebral cortex and contralateral cerebellum representing CCD.

## Introduction

Primary central nervous system lymphoma (PCNSL) is a rare neoplasm, accounting for 0.5 to 2% of all primary brain tumors and 1 to 3% of all non-Hodgkin lymphoma, with approximately 95% of PCNSLs being diffuse large B cell lymphomas (DLBCLs). PCNSL is a “whole-brain disease” from a pathological point of view, with involvement of the brain, eye, leptomeninges, and rarely spinal cord with subacute presentation in form of typical symptoms as cognitive decline or personality changes without evidence of systemic involvement. 1

The PCNSL is a vasocentric neoplasm with an infiltrative tumor extending beyond the primary lesion, with multifocality in more than 50% cases. Focal neurological deficits with involvement of the parenchyma or leptomeninges needing rapid imaging are seen in approximately 70% of the patients. 1 2

## Case Report

**Fig. 1:**
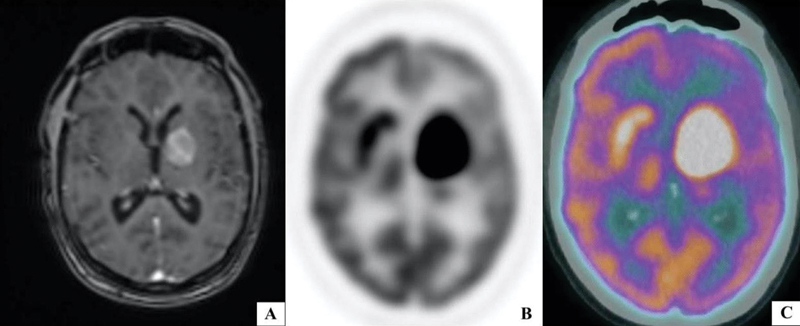
( **A** ) Gadolinium-enhanced T1-weighted MR axial and ^18^ F FDG PET and fused ^18^ F FDG PET/CT axial ( **B, C** ) showed enhancing mass in the left thalamus, internal capsule and lentiform nucleus with hypometabolism of ipsilateral parietotemporal region.

**Fig. 2:**
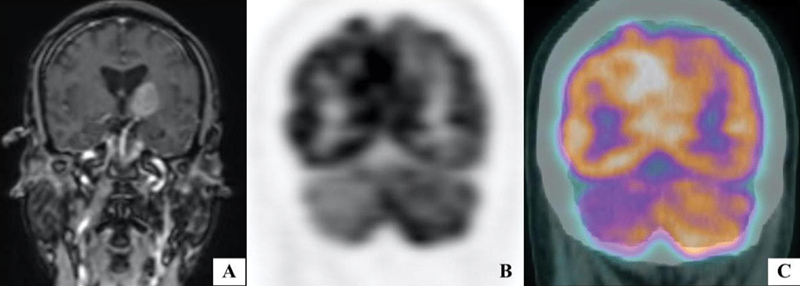
( **A** ) Gadolinium-enhanced T1-weighted MR and ^18^ F FDG PET and fused ^18^ F FDG PET/CT ( **B, C** ) coronal images showed enhancing mass in the left thalamus, internal capsule and lentiform nucleus extending into cerebral peduncle with hypometabolism of contralateral cerebellar hemisphere suggestive of crossed cerebellar diaschisis (CCD).

A 65-year-old male presented with a history of lethargy, memory loss, and hemiparesis of right lower limb. Gadolinium-enhanced T1-weighted magnetic resonance axial ( Fig. 1A ) and coronal images ( Fig. 2A ) showed enhancing mass in the left thalamus, internal capsule, and lentiform nucleus extending into cerebral peduncle.

**Fig. 3:**
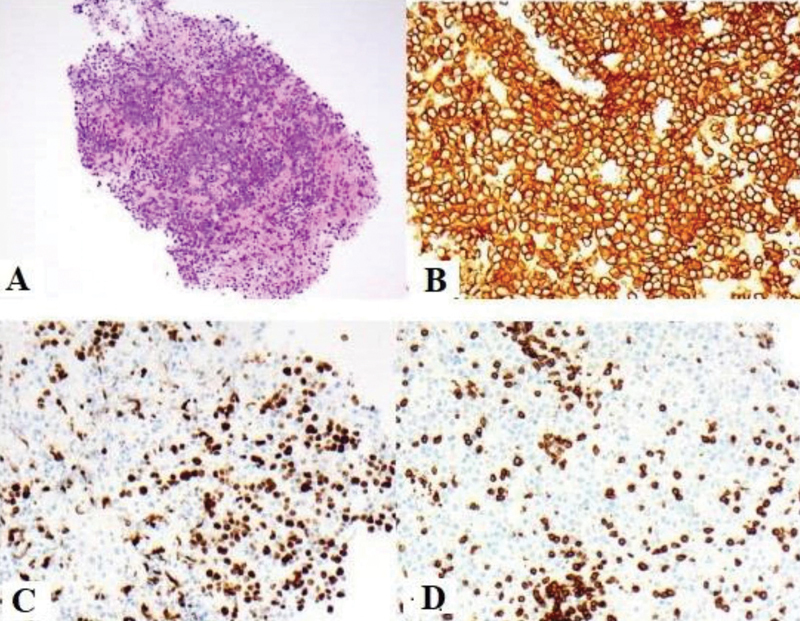
( **A** ) Biopsy showed diffuse proliferation of medium to large lymphoid cells ( **A** , H&E × 100).The neoplastic cells revealed diffuse and strong expression of CD20 ( **B** ), BCL-6, BCL-2, and MUM-1 with a very high proliferation fraction demonstrated by Ki 67 stain ( **D** ) and absent expression of CD3 ( **C** ) and CD10.

Biopsy showed diffuse proliferation of medium-to-large lymphoid cells ( Fig. 3A , hematoxylin and eosin, ×100). The neoplastic cells revealed diffuse and strong expression of CD20 (B), BCL-6, BCL-2, and MUM-1 with a very high proliferation fraction demonstrated by K _i_ 67 stain (D) and absent expression of CD3 (C) and CD10. The phenotype in combination with morphology was supportive of a diagnosis of DLBCL type of PCNSL.

^18^ F-fluorodeoxyglucose positron emission tomography ( ^18^ F-FDG PET) and fused ^18^ F-fluorodeoxyglucose positron emission tomography/computed tomography ( ^18^ F-FDG PET/CT) axial ( Fig. 1B and C ) and coronal images ( Fig. 2B and C ) showed hypermetabolic left thalamic lesion with ipsilateral hypometabolism of parietotemporal region ( Fig. 1B and C ) and contralateral cerebellar hemisphere suggestive of crossed cerebellar diaschisis (CCD). The whole body ^18^ F-FDG PET/CT imaging showed no evidence of systemic disease. The progressive imaging showed partial response to chemotherapy in the thalamic lesion.

## Discussion

The PCNSLs are commonly seen with a median age of 60 year in immune-competent patients and at a younger age in immune-compromised patients. 3 The site of PCNSL lesion determines the patients' clinical presentation. These could be focal neurological deficit signs, seizures, or neuropsychiatry symptoms as memory deficit, slowed thinking or confusion, with or without the symptoms of increased intracranial symptoms. PCNSL primarily starts with a diffuse pattern involving the deep hemispheric periventricular white matter, corpus callosum, and basal ganglia. The isolated thalamic lymphomas are a rarer cause of PCNSL, together involving the thalamus and basal ganglia. 4 The subcortical structures as the striatum are rich in mitochondria, vascular supply, neurotransmitter, and chemical content compared with other regions of the brain, making them vulnerable to metabolic anomalies and disease processes. 5 ^18^ F-FDG-PET has an important role in PCNSL staging at diagnosis or in the follow-up, as it can diagnose systemic disease with higher sensitivity than conventional imaging.

CCD is defined as decreased neuronal activity by focal structural lesions or disturbance remotely from the structures likely due to interruption of afferent and efferent pathways. CCD is a well-recognized phenomenon after cerebral infarction and reported contralateral to the focal supratentorial lesion likely due to disruption of the cortico-ponto-cerebellar tract. 6 The severity of CCD is an important prognostic marker for assessment of recovery and treatment response. 7

Vascular insult of the subcortical structures is rarely reported cause of CCD, as basal ganglia or thalamus is not usually connected to the cortico-ponto-cerebellar tract and the remote effect is usually not observed. Deep-seated thalamic infarcts have been reported to cause CCD due to their direct effect on cerebellar efferent pathways or indirect effect from the affected cerebral cortex. 8

Basal ganglia hematoma has been seen to cause CCD directly due to interruption of inhibitory GABAergic axons to globus pallidus and to thalamus through cerebellar efferent pathways resulting in reduced regional cerebral blood flow in cerebellum 9 or indirectly from interruption of dopaminergic pathways 10 or hypoperfusion of the cerebral cortex. 11 In the case of thalamic hematomas, the major anatomical pathways associated with CCD are due to the interruption of the efferent pathways from the cerebellum involving ascending cerebello-thalamo-cortical systems or due to interruption of cortico-ponto-cerebellar tract by compression of posterior limb of internal capsule or due to hypoperfusion of cerebral cortex while causing compression of cortico-ponto-cerebellar system. 8 Similar to thalamic hematomas, the mass effect due to thalamic lymphoma may cause direct or indirect interruption of the cortico-ponto-cerebellar tract and be the likely cause of CCD, although rarely reported.

Similar mass effect resulting in CCD has also been described in other brain tumors (including primary glioma), chronic subdural hematoma, congenital insults, intracranial infections, and various dementia subtypes. 6 12 13 14 15

This case is a rare demonstration of PCNS thalamic lymphoma with ipsilateral cerebral hypoperfusion and contralateral CCD likely due to the compression effect of posterior limb of internal capsule and interruption of cortico-ponto-cerebellar tract.

## Conclusion

The ^18^ F-FDG PET plays an important role in diagnosis of patients who cannot undergo brain biopsy due to surgical risks, older age, or comorbidities, with PET/magnetic resonance imaging having good accuracy for the assessment of inoperable PCNSL. 16 Whole body staging with imaging along with bone marrow biopsy to rule out the systemic diseases with secondary CNS involvement should be performed once CNS lymphoma is confirmed as outlined by International PSNSL Collaborative group. 17
